# Psychosocial adversity and the developing brain: Findings from the abcd study on 10,000 us children

**DOI:** 10.1192/j.eurpsy.2021.146

**Published:** 2021-08-13

**Authors:** S. Frangou, A. Modabbernia, G. Doucet, D. Janiri

**Affiliations:** 1 Psychiatry, University of British Columbia, Vancouver, Canada; 2 Psychiatry, Icahn School of Medicine at Mount Sinai, NEW YORK, United States of America; 3 Ageing, Neurosciences, Head-neck And Orthopaedics Sciences, Fondazione Policlinico Universitario Agostino Gemelli IRCCS, Università Cattolica del Sacro Cuore, Rome, Italy

**Keywords:** psychopathology, Neuroimaging

## Abstract

**Background:**

Childhood exposure to social risk has the potential to disrupt brain development and increase vulnerability to adverse mental health outcomes. Here, we examine the effect of adversity on brain structure and psychopathology in the Adolescent Brain and Cognitive Development (ABCD) study, a US population-based sample of 10 year-olds.

**Methods:**

Personal, caregiver, family and neighborhood characteristics were considered in 9299 unrelated children [age: mean (sd)=9.9 y (0.6); 53% males]. Hidden Markov Models were used identify clusters of participants based on their psychosocial exposure. The identified clusters were compared in terms of current psychopathology, lifetime psychiatric diagnosis, intelligence and brain structure.

**Results:**

ABCD participants clustered in to a “disadvantaged” group (N=4205) with multiple adverse exposures, and an “enriched” group (N= 5094) with limited exposure to adversity and multiple protective factors. Compared to the enriched group, the disadvantaged group had higher levels of all types of psychopathology and lifetime psychiatric diagnoses; lower scores on fluid and crystallized intelligence; smaller subcortical volumes; thinner sensorimotor cortices and thicker cortex in frontal regions; smaller surface area in temporal regions and larger surface area in the posterior cingulate cortices (all p<0.05 following Bonferroni correction for multiple testing).

**Conclusions:**

Social adversity has significant and wide-ranging consequences for brain development and psychopathology, that shows little specificity for types of symptoms.

**Disclosure:**

No significant relationships.

